# The socioecological framework for health literacy promotion in adult non-formal education: empirical findings and socioecological conceptual framework

**DOI:** 10.3389/fpubh.2026.1930078

**Published:** 2026-10-07

**Authors:** Aleksandra Bazileva, Svetlana Ušča

**Affiliations:** Center for Education, Languages and Social Technologies, Riga Technical University Rezekne Academy, Rezekne, Latvia

**Keywords:** adult non-formal education, conceptual framework, health literacy, health promotion, mixed-methods, socioecological framework

## Abstract

Rapid population aging, chronic disease prevalence, and digital information overload highlight health literacy as a critical competence for societal health promotion. This exploratory study aims to analyze theoretical dimensions of health literacy, investigate socio-demographic associations within a convenience sample of adult residents in Latvia recruited online, and synthesize these insights into a conceptual framework for health literacy promotion. A sequential explanatory mixed-methods research design was implemented in 2025/2026. Quantitative survey data (*N* = 320) were processed using IBM SPSS, conducting correlation analysis and non-parametric rank comparisons. In the qualitative phase, in-depth semi-structured interviews were conducted with key health and education sector experts (*N* = 6). Empirical results reveal a moderate positive correlation between information understanding and its practical application (*τ* = 0.383, *p* < 0.001), as well as between information accessibility and understanding in the health promotion domain (*τ* = 0.428, *p* < 0.001). Marital status was statistically significantly associated with the capacity to critically evaluate health information (*H*(3) = 10.45, *p* = 0.015). Reflexive thematic analysis (Braun and Clarke) highlighted barriers such as fragmentation in non-formal education, motivation deficits, and misinformation risks, while emphasizing employer engagement and digital health literacy. The proposed conceptual framework offers a hypothesis-generating, multi-level perspective for designing structured health literacy interventions within adult education environments.

## 1 Introduction

The rapid aging of the global and particularly European population, demographic shifts, and the rise of chronic diseases impose new demands on national sustainability strategies (1, 2). One of the most important variables in ensuring healthcare efficiency, reducing premature mortality, and promoting healthy aging is health literacy. Health literacy is no longer defined simply as an individual’s ability to read medical instructions or understand a doctor’s prescriptions; it is a complex, dynamic competence that encompasses the ability to access health information, understand it, critically appraise it, and practically apply it in daily life to maintain and improve the quality of life (3, 4).

In the context of Latvia, addressing health literacy is of paramount importance. National public health metrics indicate that mortality rates from preventable causes in Latvia remain substantially higher than the European Union average (317 per 100,000 population in Latvia compared to the EU average of 180 per 100,000) (5). Furthermore, contemporary populations face a profound systemic challenge: while rapid digitalization, telemedicine, and artificial intelligence platforms have increased the theoretical accessibility of health data, they have simultaneously catalyzed an unprecedented infodemic characterized by widespread health disinformation. This digital overload frequently generates cognitive fatigue, inappropriate self-medication practices, or detachment from evidence-based health recommendations among adults. Traditional didactic healthcare models rooted in paternalistic dynamics (“the healthcare provider instructs, the patient complies”) demonstrate limited efficacy within contemporary information environments.

To foster sustainable health behaviors, a conceptual shift in educational delivery is required. As a theoretical foundation, this study utilizes the socioecological approach derived from Bronfenbrenner’s ecological systems theory (6, 7). This perspective posits that health literacy is not an isolated individual attribute, but rather the cumulative outcome of dynamic interactions between the individual (microsystem), their immediate social environment such as family and peer networks (mesosystem), organizational and community structures (exosystem), macro-level cultural, economic, and policy frameworks (macrosystem), and temporal trajectories across the lifespan (chronosystem). Adult non-formal education, characterized by flexible delivery mechanisms and alignment with andragogical principles, represents a vital setting for implementing health literacy interventions. In non-formal adult learning, educational processes capitalize on learner autonomy, prior life experiences, and immediate practical utility, which, according to transformative learning theory, can facilitate meaningful shifts in attitudes and health practices (8, 9).

Previous health literacy research in Latvia and broader European contexts has predominantly focused on clinical environments or formal education cohorts (10, 11), leaving a distinct empirical gap regarding how adult non-formal education operates through an integrative socioecological lens (12, 13). While international evidence underscores the pivotal role of non-formal adult learning in reducing health inequities and fostering health literacy across the lifespan (3, 14), structured frameworks examining multi-level socioecological correlates in adult education environments remain underdeveloped. To address this research gap, this study aims to analyze the theoretical dimensions of health literacy within adult non-formal education, investigate its socio-demographic and environmental correlates in Latvia, and synthesize these findings into a conceptual framework for health literacy promotion.

To achieve this aim, the study addresses the following research questions:

1. What theoretical principles and empirical associations underpin health literacy development within adult non-formal education contexts?
2. What key socioecological factors and stakeholder perspectives influence the operational implementation of health literacy promotion strategies?

## 2 Theoretical framework

The theoretical foundation of this study builds upon a multi-layered synthesis of health literacy models, adult education theories, and socioecological frameworks. Historically, health literacy has been conceptualized through clinical models focusing primarily on individual cognitive and functional capabilities (11, 15). However, contemporary public health evidence emphasizes that health literacy is a dynamic, context-dependent interaction between personal competencies, non-formal learning environments, and broader socio-structural determinants (1, 5, 7).

To directly address the empirical gap regarding adult non-formal education through a socioecological lens, this conceptual framework operationalizes two interactive analytical dimensions that map directly onto the study’s research questions:

1. Theoretical Principles and Empirical Associations (RQ1): Grounded in functional, interactive, and critical literacy models (3, 11) alongside adult transformative learning theory (8, 16), this dimension synthesizes how cognitive processing, personal agency, and non-formal pedagogical environments interact to foster health literacy development.
2. Socioecological Implementation and Stakeholder Dynamics (RQ2): Utilizing socioecological frameworks (7, 12), this dimension investigates the environmental facilitators, structural barriers, and multi-stakeholder perspectives that influence the operational delivery and sustainable implementation of health literacy strategies in adult education settings.

Within an advanced andragogical framework, health literacy must be evaluated along a developmental continuum, moving from basic functionality to critical agency and empowerment. This progression operationalizes (15, 25) tripartite model, mapping directly onto Mezirow’s (8) transformative learning theory:

- *Functional Level* (Basic Tier): Foundational capacity to navigate routine clinical contexts, decode basic medical literature, and complete healthcare intake forms via direct, didactic knowledge transfer.
- *Interactive Level* (Communicative Tier): Cultivation of social competencies and cognitive self-efficacy, enabling active dialog with healthcare providers and synthesis of heterogeneous health data (17).
- *Critical Level* (Critical Agency Tier): Meta-cognitive evaluation of macro-level social, economic, and environmental determinants of health, empowering individuals to modify personal behaviors and reshape surrounding ecological structures (25).

By sequentially integrating these three developmental tiers, non-formal adult education initiatives move beyond mere informational transmission, purposefully guiding adult learners from functional compliance to critical reflection and critical health literacy.

To maintain focus on the core analytical framework and empirical design, the detailed taxonomy of health literacy criteria across cognitive domains and the comparative synthesis of adult education principles (23, 24) are transferred to the Supplementary Tables S1, S2.

## 3 Materials and methods

This study utilized a sequential explanatory mixed-methods design (QUAN → qual). In the first phase, quantitative survey data (*N* = 320) were collected to establish baseline health literacy profiles and identify socio-demographic associations. In the second phase, qualitative semi-structured expert interviews (*N* = 6) were conducted to contextualize the quantitative results, explore systemic barriers within adult education, and gather expert perspectives. Data integration occurred at two points: (1) quantitative findings informed the qualitative interview protocol design, and (2) quantitative and qualitative results were jointly synthesized during narrative integration to develop the proposed conceptual framework.

### 3.1 Research phases and sampling strategy

#### 3.1.1 Quantitative phase

The quantitative data collection was executed between February and November 2025 via a digital cross-sectional survey. The survey instrument utilized a health literacy questionnaire adapted and refined by the authors, operationalizing the core dimensions of the internationally validated HLS-EU-Q47 instrument. The original 47-item instrument was translated into Latvian using a formal forward-backward translation protocol. Items were contextually adapted and pre-tested via a pilot study (*n* = 11) to confirm cognitive clarity and semantic validity. Participant recruitment was executed via an open-access, non-probability voluntary sampling strategy. The survey was disseminated digitally across social media networks, primarily utilizing the lead author’s public profile and public thematic groups on Facebook. Due to the open nature of social media distribution and the lack of a defined initial sampling frame (denominator), a formal response rate cannot be computed. To ensure dataset integrity, the online survey architecture incorporated forced-choice response validation logic, requiring respondents to complete all items on a given page before proceeding to the next section. Incomplete entries were automatically terminated by the system upon user exit prior to final submission. Consequently, the final analytical sample (*N* = 320) comprised fully completed questionnaires with zero missing data across all operationalized variables. Crucially, the quantitative survey captured general adult residents and did not measure whether respondents were currently or previously enrolled in adult non-formal education programs.

#### 3.1.2 Qualitative phase

To deeply investigate the identified barriers and socioecological determinants of health literacy, semi-structured, online video interviews (lasting 45–60 min each) were conducted with key sectoral experts (*N* = 6), including public health professionals, educational specialists, and institutional policymakers. Expert participants were recruited via purposive sampling based on the following pre-established inclusion criteria:

- Possession of a relevant higher education degree within health sciences, pedagogy, or public administration;
- Active professional tenure and residence in Latvia (minimum of 5 years), ensuring direct institutional experience and deep familiarity with Latvia’s specific healthcare, educational, and sociopolitical infrastructure;
- Direct institutional competence in adult non-formal education delivery or national public health policy implementation.

The generated qualitative data were processed and analyzed using reflexive thematic analysis, allowing for the systematic structuring of transcripts into defined thematic nodes by the methodological framework of Braun and Clarke (18, 19).

#### 3.1.3 Data integration protocol

Data integration followed a sequential explanatory joint-display protocol (QUAN → qual). Quantitative outcomes (socio-demographic variations in health literacy dimensions) directly dictated the qualitative inquiry matrix. Qualitative findings from expert interviews were then mapped side-by-side against specific quantitative results to evaluate contextual convergence, offer mechanistic explanations (e.g., family structures as cognitive filters), and identify structural divergences within adult non-formal education.

### 3.2 Quantitative instrument validation, pilot testing, and statistical analysis

To evaluate the psychometric reliability, contextual clarity, and formatting accessibility of the adapted survey instrument, a preliminary pilot administration was conducted on December 7, 2024 (*n* = 11) as a non-invasive methodological pre-test to verify instrument syntax. Following formal institutional ethical clearance on February 18, 2025, the primary survey data collection commenced. Participants reported no substantive difficulties in understanding or navigating the questionnaire. Internal consistency analysis of the pilot instrument yielded a Cronbach’s alpha coefficient of *α* = 0.971, demonstrating high internal consistency. Data collected during the pilot phase were strictly excluded from the main analytical sample (*N* = 320). Within the main analytical dataset (*N* = 320), the adapted survey instrument demonstrated high overall internal reliability, with a Cronbach’s alpha coefficient of *α* = 0.926.

Quantitative data curation and computational analyses were executed using IBM SPSS Statistics and Microsoft Excel. All statistical descriptions and analytical procedures were consolidated into this section to avoid redundancy. Exploratory data analysis via the Kolmogorov–Smirnov test revealed that 37 out of 47 items significantly deviated from a normal distribution (*p* < 0.05). Given this non-normality, a non-parametric inferential statistical framework was applied:

- Kendall’s *τ* rank correlation coefficients. Deployed to evaluate the direction, monotonicity, and strength of inter-relationships between discrete health literacy criteria.
- Kruskal–Wallis *H* test: Deployed to identify statistically significant differences across more than two independent socio-demographic categories.
- Mann–Whitney *U* test: Applied for pairwise comparative analyses between two independent cohorts.

All non-parametric tests were exploratory in nature. Because residual data constraints prevented fitting robust multivariable regression models across ordinal sub-scales *post hoc*, all reported quantitative associations represent unadjusted bivariate comparisons. Unadjusted multiple comparisons carry a potential risk of inflated Type I errors; thus, findings are interpreted as exploratory statistical associations rather than definitive causal claims. In total, 25 bivariate non-parametric comparisons were performed across the socio-demographic variables and health literacy dimensions.

Given the convenience sampling approach (*N* = 320, predominantly female and university-educated), all statistical comparisons are interpreted as exploratory and hypothesis-generating. Residual demographic collinearity and unmeasured confounding variables (such as age, underlying digital literacy, or health status) cannot be fully controlled within these bivariate models.

### 3.3 Ethical considerations

This study was conducted in strict compliance with the fundamental ethical principles of scientific research and the General Data Protection Regulation (GDPR). Institutional ethical clearance was independently obtained and validated for both empirical phases prior to their respective data collection timelines:

- *Quantitative Phase Approval*: institutional ethical clearance for the survey administration was granted by the Council of the “Education, Pedagogy and Sports” study direction at the Rezekne Academy of Technologies on February 18, 2025 (Protocol No. 6, Decision No. 1.1), in accordance with the institutional methodological guidelines for scientific research.
- *Qualitative Phase Approval*: following the institutional restructuring and integration into the Riga Technical University framework, the qualitative research protocol, expert interview instruments, and informed consent materials were formally reviewed and approved by the Council of the Center for Education, Languages and Social Technologies at Riga Technical University Rezekne Academy on January 26, 2026 (Protocol No. 4).

All research participants (including survey respondents and qualitative expert interviewees) were thoroughly briefed on the research objectives, data utilization protocols, anonymization procedures, and strict confidentiality guarantees before enrollment. Participation was entirely voluntary, validated by obtaining electronic or written informed consent prior to data elicitation. All data streams were processed and analyzed in an aggregate, fully anonymized format.

## 4 Results

### 4.1 Sample Characteristics

The socio-demographic characteristics of the quantitative survey sample (*N* = 320) are detailed in Table 1.

**Table 1:** Socio-demographic characteristics of the survey respondents (*N* = 320).

Demographic variable	Category	*N*	%
Sex	Female	293	91.6
	Male	27	8.4
Age group	18–29 years	45	14.1
	30–44 years	139	43.4
	45–59 years	108	33.8
	≥ 60 years	28	8.8
Educational attainment	Basic Education	3	0.9
	Secondary/vocational	85	26.6
	Bachelor’s degree	119	37.2
	Master’s degree	110	34.4
	Doctoral degree	3	0.9
Occupational status	Public/municipal sector employee	186	58.1
	Private sector employee	79	24.7
	Student	16	5.0
	Unemployed	13	4.1
	Entrepreneur/self-employed	10	3.1
	Homemaker	8	2.5
	Retired/pensioner	8	2.5
Marital status	Married/cohabiting	210	65.6
	Single	71	22.2
	Divorced/separated	26	8.1
	Widowed	13	4.1
Parental status	With children	247	77.2
	Without children	73	22.8

### 4.2 Correlation analysis and systemic interaction of health literacy metrics

The descriptive statistical analysis revealed that the global mean health literacy index within the study sample (*N* = 320) was 33.45 ± 9.41 points (*M* = 33.45, SD = 9.41; Median = 33.83, Mode = 35.67). Evaluated against the standardized HLS-EU metric thresholds (11), where 0–25 points denote inadequate, >25–33 problematic, >33–42 sufficient, and >42–50 excellent health literacy. Respondents were distributed across categories as follows: inadequate (*n* = 38, 11.9%), problematic (*n* = 104, 32.5%), sufficient (*n* = 118, 36.9%), and excellent (*n* = 60, 18.7%). While the mean index borders the problematic and sufficient thresholds, applying standardized threshold bands to an adapted survey instrument within a non-probability convenience sample warrants methodological caution, as categorical boundaries may not fully capture sample-specific contextual nuances.

Bivariate correlation analysis across four primary sub-metrics (accessing information (ACC), understanding information (UND), appraising information (APP), and applying information (APY)) yielded positive and statistically significant coefficients (*p* < 0.01). In alignment with standardized statistical reporting, Kendall’s *τ* = 0.383 is classified as a moderate positive correlation across all sections.

As shown in Table 2, the correlation between information accessibility (ACC) and understanding (UND) specifically within the health promotion domain was statistically significant (*τ* = 0.428, *p* < 0.001).

**Table 2:** Kendall’s *τ* correlation coefficients between health literacy dimensions.

Variable pair	Domain	Correlation coefficients (*τ*)	*p*-value
ACC vs. UND	Health promotion	0.428	<0.001
UND vs. APY	Overall	0.383	<0.001
ACC vs. APP	Overall	0.312	<0.001
ACC vs. UND	Overall	0.272	<0.001
APP vs. APY	Overall	0.232	<0.001

### 4.3 Socio-demographic variations in health literacy

Inferential statistical modeling revealed several statistically significant non-parametric associations across socio-demographic groups.

#### 4.3.1 Educational attainment

The Kruskal–Wallis test demonstrated no statistically significant differences in the global health literacy index or within the health promotion (HP) dimension across levels of educational attainment (*H*(4) = 4.12, *p* = 0.390, *η*^2^ = 0.003). However, a statistically significant variance was identified regarding the practical application of information (APY) (*H*(4) = 11.24, *p* = 0.024, *η*^2^ = 0.023), with higher mean ranks observed among respondents with Master’s (mean rank = 172.65, *n* = 110) and Doctoral degrees (mean rank = 175.50, *n* = 3) compared to Bachelor’s degrees (mean rank = 158.40, *n* = 119), secondary education (mean rank = 142.10, *n* = 85), and basic education (mean rank = 63.50, *n* = 3). Crucially, because the basic education (*n* = 3) and doctoral degree (*n* = 3) cells contain very small sample sizes, mean-rank comparisons across these five categories are uninterpretable at the extremes and must be regarded as strictly exploratory.

#### 4.3.2 Marital status

Evaluating differences across marital status categories regarding the capacity to critically appraise health information (APP) revealed a statistically significant inter-group variance (*H*(3) = 10.45, *p* = 0.015, *η*^2^ = 0.024). Married respondents (*n* = 210) exhibited the highest mean rank (171.52), followed by widowed (*n* = 13, mean rank = 146.88), single (*n* = 71, mean rank = 140.26), and divorced individuals (*n* = 26, mean rank = 133.58).

#### 4.3.3 Occupational status

The Kruskal–Wallis test isolated a statistically significant inter-group difference exclusively within the information access (PI) dimension of healthcare (*H*(6) = 13.18, *p* = 0.007, *η*^2^ = 0.023). The highest mean ranks were identified within the student cohort (*n* = 16, mean rank = 191.28) and among public/municipal sector employees (*n* = 186, mean rank = 174.51), Lower mean ranks were recorded among self-employed entrepreneurs (*n* = 10, mean rank = 157.35), retired individuals (*n* = 8, mean rank = 155.88), private sector employees (*n* = 79, mean rank = 132.46), homemakers (*n* = 8, mean rank = 130.56), and unemployed individuals (*n* = 13, mean rank = 116.23). Within the remaining healthcare dimensions, information understanding, information evaluation, and information application, occupational category demonstrated no statistically significant variance (*p* = 0.556, *p* = 0.450, and *p* = 0.453, respectively).

#### 4.3.4 Parental status

The Mann–Whitney *U* test indicated that the presence of children within the household significantly differentiated competencies across two distinct phases: accessing information for disease prevention (*U* = 7,245.0*, p* = 0.012, *r* = 0.14) and understanding information for health promotion (*U* = 7,482.5, *p* = 0.038, *r* = 0.12). In both instances, respondents without children (*n* = 73) exhibited significantly higher scores (mean ranks of 184.01 and 179.66, respectively) than respondents with children (*n* = 247; mean ranks of 153.55 and 154.84).

#### 4.3.5 Gender

Due to substantial sample imbalance between female (*n* = 293) and male respondents (*n* = 27), gender comparisons are severely underpowered and strictly exploratory. Mann–Whitney U tests revealed no statistically significant differences between genders across primary health literacy competencies (*p* > 0.05). A non-significant statistical trend emerged regarding the practical application of health promotion information (*U* = 3,210.5, *p* = 0.089, *r* = 0.09), with female respondents demonstrating a higher mean rank (163.14) than male respondents (131.81).

### 4.4 Qualitative findings from sectoral experts: barriers and promotional resources

A reflexive thematic analysis of the expert interview transcripts isolated critical systemic barriers and innovative resources for promoting health literacy. These findings were synthesized into three primary conceptual nodes:

#### 4.4.1 Node 1: pedagogical mechanisms in adult non-formal education

- Barriers: Experts emphasized that because adult non-formal education operates on a voluntary enrollment basis, the primary structural obstacle is a deficit in initial motivation. The public frequently seeks educational interventions reactively, only when an acute health crisis or disease manifests. Furthermore, significant risks regarding educator competence were identified, notably the unvalidated proliferation of “alternative theories” within non-formal courses.

“Since education is not compulsory, it depends heavily on the individual’s desire to educate themselves… The proficiency level of the lecturers is critical, meaning who teaches, who is being taught, and what is being taught. This process must not be surrendered to incompetent individuals.” (Expert 1, Healthcare Specialist).

- Solutions: Experts called for an immediate paradigm shift toward advanced andragogical methodologies grounded in a socioecological framework, combined with the strategic marketing of professional programs to proactively engage the healthy population.

“Lecturing are the least effective format… An individual must be engaged at both cognitive and physical levels through seminar-style sessions and active discussions.” (Expert 4, Education Specialist).

#### 4.4.2 Node 2: socioecological factors and environmental dynamics

- Barriers and Social Networks: Regarding macro-systemic policy, multiple experts explicitly stated that state-level policy modulates public access to high-quality knowledge “by 200%,” yet a critical lack of institutional centralization persists. Additional threats include aggressive digital disinformation pressure and the socioecological inheritance of obsolete generational stereotypes, where family networks operate as a powerful cognitive filter that obstructs the adoption of evidence-based health practices. On one hand, outdated intergenerational practices can obstruct the adoption of evidence-based health choices. On the other hand, experts emphasized that close interpersonal networks function as an essential cognitive and experiential anchor, explicitly noting that “when seeking health information, people rely on the insights, knowledge, and experience of relatives and colleagues” (Expert 1, Healthcare Specialist). Thus, supportive spousal, family, and workplace dynamics within the mesosystem operate as a buffer, enabling shared health decision-making and collaborative filtering of health information.

“Policy influences access to knowledge by 200 percent… Everything depends on the political stance; there must be some form of centralization.” (Expert 2, Political Specialist).

“People express views inherited from their parents that are simply obsolete… What worked 20 or 30 years ago does not really work now…especially in burn cases, where I see people applying sour cream or other inappropriate substances. When you explain that this should not be done, they do not listen, explaining that grandmother taught them so.” (Expert 1, Healthcare Specialist).

- Resources: To counter these barriers, experts highlighted the imperative for centralized state support and robust content validation mechanisms implemented in close collaboration with the medical community.

“Shifting the health literacy promotion process into the hands of the state.” (Experts 2 and 5, Political Specialists).

#### 4.4.3 Node 3: innovative resources and implementation efficacy

Three primary strategic pathways emerged as highly engaging, novel channels for systemic health literacy optimization: active employer engagement (integrating wellness programs within corporate environments, incentivizing organizations via targeted tax reliefs), community-anchored spaces for demographics outside the active labor force, and intergenerational learning models. While corporate infrastructures serve as key exosystemic drivers for the employed population, experts emphasized the imperative to decentralize learning settings to reach homemakers and retirees who face spatial and institutional isolation from traditional workplace-based initiatives.

“There are various organizations and associations where non-formal health literacy education could be introduced through interest-based learning. We have quite a few such associations, for example, seniors’ associations, state employment agencies, mothers’ clubs… Thus, working with the community through specific target groups.” (Expert 2, Political Specialist).

Finally, intergenerational learning models emerged as a highly promising mechanism to bypass time and cognitive load constraints experienced by parents. Qualitative findings drew a distinct boundary between an individual’s cognitive health literacy capacity and their practical daily prioritization. Experts explicitly underscored the acute time limits faced by parents, highlighting: “*In general, with children, we cover foundational health topics (hygiene, nutrition, physical activity, and healthy habits) and that is our responsibility because parents lack time resources*” (Expert 4, Education Specialist). Lower engagement in personal health information seeking among parents does not reflect a deficit in information comprehension capacity, but rather acute caregiver time scarcity and competing household demands, wherein parents systematically prioritize their children’s acute needs over their own proactive health promotion. Consequently, child-centered educational initiatives function as a bi-directional vector, indirectly elevating adult health literacy through domestic knowledge transfer without imposing additional time burdens on busy parents.

“…it is essential to include the child population because… health topics must be introduced from the preschool period so that this knowledge continuously develops and refines over time. Furthermore, parents learn through their children.” (Experts 4 and 6, Education Specialists).

“In practice, adult engagement occurs through an indirect connection, interacting through the children’s parents. We could design educational materials that we, as preschool staff, could offer to the children, so that parents learn alongside them.” (Expert 6, Education Specialists).

In summary, the solutions identified by sectoral experts delineate a necessary paradigm shift from reactive crisis management toward a proactive, systemically integrated framework for health literacy promotion. The successful execution of this educational ecosystem depends on building strategic synergy among state governance, corporate employers, and the medical community.

As synthesized in Table 3 via a joint-display protocol, the qualitative expert insights directly contextualize and unpack the statistical variances identified in the quantitative survey phase, establishing methodological triangulation.

**Table 3:** Joint display integrating quantitative findings with qualitative expert contextualization.

Quantitative finding (QUAN)	Statistical metric	Qualitative contextualization (qual)	Integration outcome
Mesosystemic variance (marital status): married respondents demonstrate higher scores in critical appraisal (APP).	*H*(3) = 10.45, *p* = 0.015, *η*^2^ = 0.024	Spousal cognitive filter: experts highlighted that family networks enable shared decision-making, internal dialog, and collaborative filtering of digital disinformation.	Convergence: qualitative data explain quantitative variance via mesosystemic social support mechanisms.
Exosystemic variance (occupational status): students and public sector employees show higher healthcare information access.	*H*(6) = 13.18, *p* = 0.007, *η*^2^ = 0.023	Institutional access: public/corporate environments offer structured wellness info, whereas homemakers face isolation from non-formal learning spaces.	Expansion: explains why workplace infrastructure serves as a primary exosystemic driver for health literacy.
Parental status variance: respondents without children score higher in accessing and understanding health promotion data.	*U* = 7,245.0*, p* = 0.012, *r* = 0.14	Caregiver prioritization vs. literacy capacity: experts emphasized that parents face competing daily demands, noting that *“parents lack time resources”* and prioritize immediate child healthcare needs over personal proactive health information seeking.	Contextualization: disentangles practical behavioral prioritization and time scarcity from cognitive health literacy capacity, showing that lower engagement reflects caregiver bandwidth allocation rather than comprehension deficits.
Internal dimension correlation: moderate correlation between understanding (UND) and applying (APY) health data.	*τ* = 0.383, *p* < 0.001	Andragogical paradigm: experts emphasized that passive lecturing fails; active comprehension is required for behavioral application.	Confirmation: reinforces adult learning theory within the proposed conceptual framework.

To systematically operationalize and visualize these empirical insights, a holistic, multi-dimensional conceptual framework was developed: the Socioecological Framework for Health Literacy Promotion in Adult non-formal Education (see Figure 1). This conceptual framework serves as a comprehensive methodological blueprint for designing future adult non-formal education systems.

**Figure 1:**
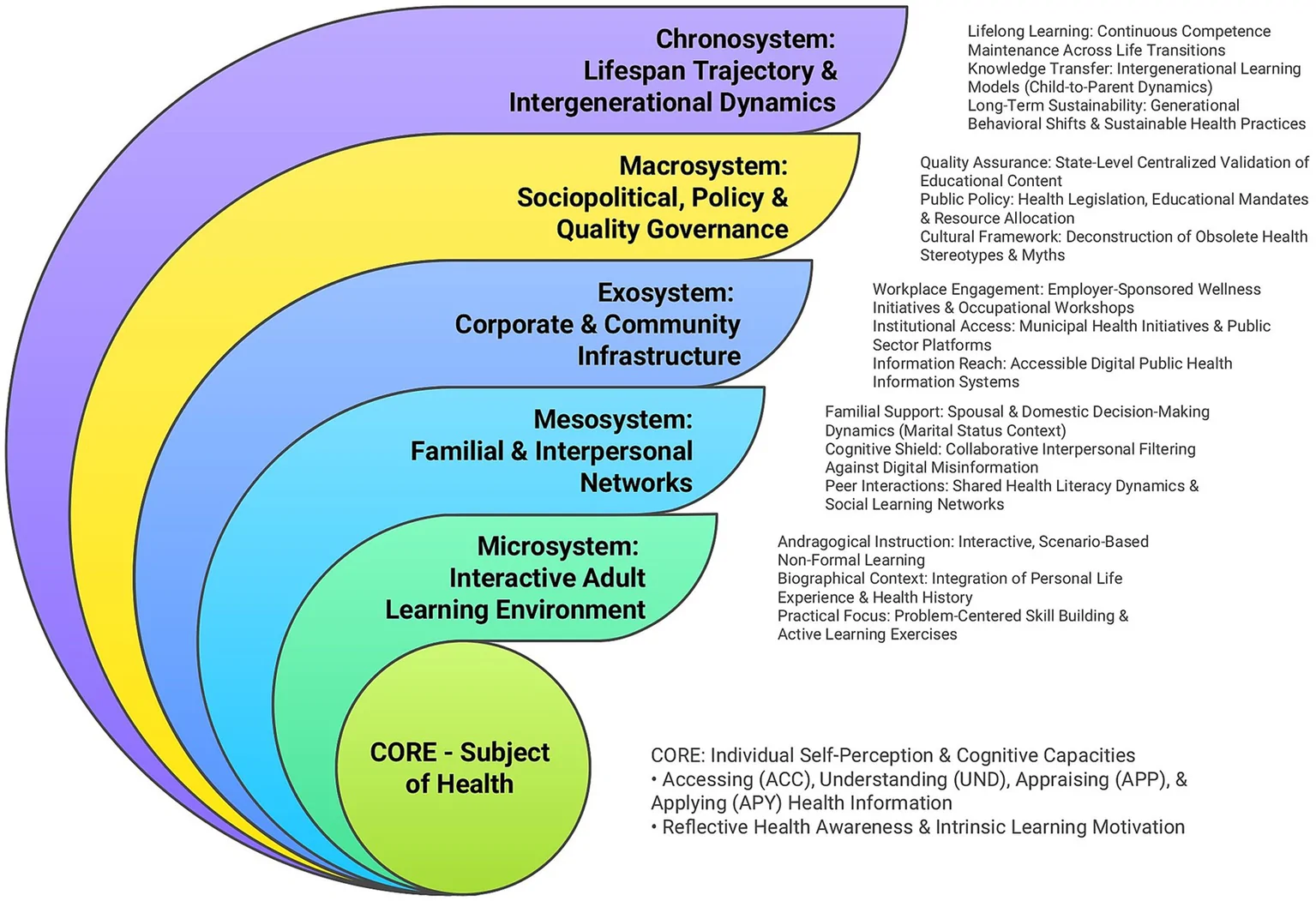
Socioecological framework for health literacy promotion in adult non-formal education.

Structural Breakdown of the Conceptual Framework Components:

- *The Core—Subject of Health*. The nucleus of the framework isolates the individual as an active, agentic subject at the nexus of conscious and autonomous decision-making. Health literacy promotion originates within the foundational personal layer through individual health self-perception, reflective awareness, and self-directed health information processing. At this juncture, the core cognitive dimensions of health literacy (accessing, understanding, appraising, and applying health information) materialize, translating knowledge into verifiable health-promoting behaviors.
- *The Microsystem*. This layer encompasses the immediate learning and social environments inhabited by the adult learner, incorporating personal biographical experience, active problem-solving scenarios, and direct non-formal educational settings. Grounded in andragogical principles, primary health habits and intrinsic learning motivation are fostered at this level through interactive instructional engagement.
- *The Mesosystem*. Representing the structural linkages between immediate microsystems, this layer positions spousal networks, immediate family structures, and close peer groups as vital social supports. Reflected in empirical findings on marital status, collaborative evaluation and domestic decision-making are hypothesized to support critical health data appraisal, potentially functioning as interpersonal buffers against digital misinformation and pseudoscientific claims.
- *The Exosystem*. This dimension encompasses institutional environments and indirect settings that structurally modulate learning opportunities. As demonstrated by empirical occupational variances and qualitative expert insights, the corporate work environment serves as a key exosystemic intervention setting. Integrating structured workplace wellness programs and accessible digital public health resources provides organized channels for adult health education.
- *The Macrosystem*. This overarching layer embodies macro-level sociopolitical, legal, and cultural frameworks. It encompasses state public health policy, centralized course validation standards, public health resources, and broader societal health beliefs. Establishing centralized quality standards for non-formal adult education and addressing outdated cultural stereotypes create the structural foundation for population-wide health literacy promotion.
- *The Chronosystem*. Encompassing the temporal dimension, the chronosystem intersects all preceding layers, framing health literacy as a dynamic, lifelong learning process across life transitions. It integrates intergenerational learning dynamics (child-to-parent knowledge transfer), facilitating long-term behavioral sustainability and updating generational health practices over time.

The operational mechanics of health literacy promotion within this framework are distinctly non-linear; rather, they are structural, circular, and synergistic. Macro-systemic political mandates and exosystemic corporate infrastructures activate and resource the mesosystem. The mesosystem, in turn, directly stimulates individual cognitive capacities and reflective health awareness, thereby achieving sustainable, long-term public health behavioral shifts across the temporal spectrum of the chronosystem.

## 5 Discussion

The empirical findings generated in this study indicate that the promotion of health literacy within Latvian society cannot be achieved via superficial, transient informational campaigns, such as the dissemination of pamphlets or static posters.

### 5.1 Correlation of internal health literacy metrics and the educational paradox

The statistical analysis demonstrates that among the overall operational dimensions, a key positive correlation exists between the understanding of health information and its practical application (*τ* = 0.383, *p* < 0.001), while the domain-specific association between information accessibility and comprehension in health promotion reached *τ* = 0.428 (*p* < 0.001). This is a critical finding, empirically supporting the notion that the mere mechanical transmission of data or the superficial provision of information accessibility is not autonomously associated with health-promoting behaviors. Instead, deep and unambiguous comprehension demonstrates a moderate positive correlation with an adult learner’s capacity to modify their behavioral patterns and effectively apply health knowledge in daily practice. This conclusion aligns with the integrated health literacy matrix conceptualized by Sørensen et al. (11) and reinforces recent systematic evidence by Gonçalves-Fernández and Pino-Juste (1), who demonstrated that health literacy in healthy adult populations relies fundamentally on higher-order cognitive processing and practical comprehension rather than passive access to information.

Furthermore, within the health promotion dimension, a significant positive correlation was identified between information accessibility and comprehension (*τ* = 0.428, *p* < 0.001), which underscores the imperative for high-quality, reliable, and linguistically accessible primary information sources. Consequently, the instructional architecture of adult non-formal education must pivot away from rote data reproduction toward the cultivation of advanced critical appraisal skills and deep conceptual understanding.

In this context, the relationship between formal education and health literacy appears domain-specific rather than uniform. While global health literacy index scores and health promotion competencies showed no statistically significant variance across educational levels (*p* = 0.390), higher formal education was specifically associated with higher mean ranks in the practical application of health data (APY, *p* = 0.024). This nuanced pattern suggests that general academic training provides foundational analytical tools that may support application skills, yet overall health literacy encompasses broader, context-dependent capabilities that do not automatically scale with formal degree attainment. Nevertheless, because extreme educational categories (basic education and doctoral degree) comprised very small cell sizes (*n* = 3 in each), these subgroup differences remain exploratory and cannot support definitive conclusions regarding an overarching educational paradox (5, 12, 20).

### 5.2 Mesosystemic influences and social support systems

A particularly compelling finding pertains to the statistically significant variance observed across interpersonal support structures (operationalized via the respondent’s marital status within the mesosystem) in the capacity to critically appraise health information (*p* = 0.015). This statistical association aligns conceptually with Urie Bronfenbrenner’s socioecological framework (6, 7), suggesting that mesosystemic dynamics (spousal and familial support, collective decision-making, and internal dialog) may function as interpersonal resources associated with an individual’s capacity to navigate complex information streams and exercise critical judgment.

While traditional adult education methodologies remain predominantly individual-centric, these findings suggest superior efficacy in pedagogical programs that promote shared familial participation or provide materials designed for domestic collective review. Social support within familial mesosystemic networks is hypothesized to function not merely as an emotional buffer, but as an interpersonal cognitive filter that may assist individuals in critically evaluating health information and navigating digital disinformation risks. Similar conclusions regarding the role of the family as a cohesive health literacy community are advanced by Edwards et al. (21) and supported by Seetharaman et al. (9), who emphasize that health-related decision-making and adult transformative learning outcomes are fundamentally relational and collective rather than isolated individual phenomena.

The observed statistical variance regarding parental status requires careful theoretical interpretation to avoid conflating cognitive capability with situational behavior. As highlighted by the qualitative contextualization, lower engagement in accessing disease prevention data and understanding personal health promotion information among parents reflects a shift in daily prioritization and cognitive bandwidth allocation rather than a systemic deficit in functional health literacy capacity. Under the framework of cognitive load theory and family decision-making models (3, 4), adult caregivers under heavy time scarcity prioritize acute dependent needs (child healthcare and immediate family welfare) over proactive personal health seeking. Thus, practical prioritization in everyday life operates independently from an individual’s underlying ability to process health data, illustrating how competing social roles within the microsystem temporarily suppress proactive health promotion engagement.

### 5.3 Exosystemic and macrosystemic frameworks: the corporate environment and intergenerational learning

The strategic integration of corporate employers (the exosystem), as proposed by the sectoral experts, represents a highly innovative intervention pathway within the context of Latvian public health. The workplace constitutes a primary source of chronic stress and epidemiological risks for the adult population, a reality reflected in the quantitative data regarding the statistically significant variance across occupational status categories in healthcare information access (*H*(6) = 13.18, *p* = 0.007, *η*^2^ = 0.023). As highlighted by Bano and Hou (16), adult learning engagement is heavily modulated by environmental context and situational relevance; thus, embedding non-formal health education within familiar corporate settings may help address adult engagement barriers and foster sustained participation.

If macro-systemic governance could institutionalize targeted economic incentives (such as structural tax reliefs for enterprises that co-finance or fully subsidize non-formal health education for their workforce) this would facilitate mass, systemic access to the adult target demographic directly within the corporate environment. This framework directly aligns with the World Health Organization’s directives on health-promoting workplace development (22), effectively transforming the corporate exosystem into a supportive environment for health literacy optimization.

Concurrently, the deployment of an intergenerational learning model (transmitting literacy dynamics from children to parents) offers a sustainable mechanism to disrupt deeply entrenched macro-systemic stereotypes and the curative-centric paradigm (“the cult of treatment”) embedded within the Latvian cultural space. As proposed by experts in the qualitative phase, integrating children into health literacy initiatives represents a hypothesized pathway for encouraging broader family engagement and indirect knowledge transfer.

### 5.4 Practical and policy implications for adult non-formal education

To translate these empirical insights and the proposed socioecological framework into actionable practice, several strategic recommendations are outlined for adult education providers, healthcare institutions, and decision-makers in Latvia:

1. *Andragogical Redesign of Learning Curricula*: adult non-formal education providers should transition from passive informational lecturing toward interactive, scenario-based learning formats. Curricula should prioritize critical information appraisal (e.g., evaluating online source credibility, recognizing digital health misinformation) and practical health application rather than rote data transmission.
2. *Workplace Infrastructure Integration (Exosystemic Pathways)*: employers and municipal institutions should embed modular health literacy workshops directly into organizational wellness initiatives. Policymakers should explore economic incentives, such as tax relief or co-financing mechanisms, to encourage corporate investment in adult health education.
3. *Family-Centric and Intergenerational Interventions (Mesosystemic Pathways)*: educational programs should be designed to engage adult learners alongside family members or through intergenerational models (leveraging child-to-parent knowledge transfer), capitalizing on spousal and familial support networks as protective cognitive filters against health misinformation.
4. *State-Level Quality Assurance and Content Centralization*: public health authorities, in collaboration with professional medical associations, should establish centralized quality standards and validation mechanisms for non-formal adult health education courses to mitigate the risks of unverified pseudoscientific content.

## 6 Strengths and limitations

This study possesses several notable strengths, including the application of a sequential explanatory mixed-methods design that combines quantitative survey metrics with in-depth qualitative expert insights, anchored in a well-established socioecological paradigm. Furthermore, the focus on non-formal adult education addresses an under-researched setting in health literacy literature.

However, several methodological limitations must be acknowledged:

1. *Cross-Sectional and Unadjusted Bivariate Design*: The quantitative data are cross-sectional, precluding any causal inferences or directionality. Furthermore, quantitative comparisons were based on unadjusted bivariate non-parametric tests without multivariable adjustment for potential confounders (e.g., age or income) or adjustment for multiple testing. Consequently, findings reflect unadjusted exploratory associations and should be interpreted with caution.
2. *Sampling and Selection Bias*: Data were gathered via convenience online sampling among adult residents in Latvia (*N* = 320), which likely overrepresented individuals with higher baseline digital literacy and higher formal education levels (72.5% holding university degrees). Adults with limited digital access, lower functional health literacy, or marginalized socioeconomic status may be underrepresented. Furthermore, participation in adult non-formal education was not operationalized or measured in the quantitative survey. Consequently, the quantitative strand reflects general adult population associations, whereas empirical insights regarding adult non-formal education as a specific instructional setting deriving exclusively from the qualitative expert interviews.
3. *Qualitative Sample Size*: Qualitative findings were derived from six key sectoral experts. While expert interviews yielded rich contextual data, expanding participant representation to include adult learners directly would enhance ecological validity in future research.
4. *Conceptual Framework Boundaries*: The proposed socioecological framework represents an empirically informed conceptual mapping rather than an empirically tested structural model. Future studies should employ structural equation modeling (SEM) or longitudinal intervention designs to formally evaluate hypothesized pathways.

## 7 Conclusion

Within contemporary adult education contexts, health literacy operates as a dynamic, multi-layered competence developing through continuous interactions between the individual and their socioecological environment. Findings from this exploratory cohort offer hypothesis-generating baseline insights regarding adult health literacy engagement in non-formal learning settings in Latvia.

The promotion of health literacy possesses a distinctly pedagogical character, within which the programmatic methodological framework and the instructor’s professional competence play a decisive role. Furthermore, low public participation rates may be attributed to prior negative experiences with low-quality courses (as isolated by sectoral experts) compounded by insufficient targeting that fails to align with the practical interests of adult learners.

Systemic health literacy promotion is theoretically grounded in a dual-framework: the socioecological systems theory, which defines the multi-layered environment (micro- to chronosystem), and transformative learning theory, which provides the cognitive mechanism (critical reflection and paradigm shift) necessary to move adult learners from functional compliance to critical health literacy.

This mixed-methods empirical study in Latvia identified positive bivariate correlations among health literacy dimensions, observing a moderate positive correlation between the comprehension of health information and its practical application in daily routines (*τ* = 0.383, *p* < 0.001).

The generated data provide preliminary empirical insights regarding mesosystemic influences, revealing that the respondent’s marital status is statistically significantly associated with variance in the capacity to critically appraise health information (*p* = 0.015). Married individuals demonstrated higher critical appraisal rank scores, suggesting that supportive social and familial environments within the mesosystem may correlate with enhanced cognitive resilience against digital disinformation.

The qualitative content analysis of sectoral experts highlighted a need for the centralized validation of non-formal education content and macro-level political support. Concurrently, active employer engagement at the exo-level and intergenerational learning models at the meso-level were identified as highly engaging, hitherto underutilized promotional resources. To operationalize these findings, adult non-formal education providers and policymakers in Latvia should prioritize interactive andragogical formats, institutionalize workplace health literacy initiatives, leverage familial mesosystemic support, and establish state-level quality assurance for educational content.

## Data Availability

The raw data supporting the conclusions of this article will be made available by the authors, without undue reservation.
