# Supplementary material for: The socioecological framework for health literacy promotion in adult non-formal education: empirical findings and socioecological conceptual framework
Source: Front Public Health. 2026 Oct 7;14:1930078. doi: 10.3389/fpubh.2026.1930078 (PMC13683574; doi:10.3389/fpubh.2026.1930078)
Supplement: Supplementary file 1 [file Data_Sheet_1.pdf]

Supplementary Material

Table S1

Health literacy criteria across cognitive processes and application domains (adapted from Sørensen et al., 2012)

Domain of application	Cognitive dimension (process)	Operational explanation
Healthcare	Accessing information	The capacity of an individual to retrieve information regarding clinical symptoms, therapeutic modalities, and avenues for professional healthcare services.
	Understanding information	The capacity to comprehend and interpret clinical advice, pharmaceutical instructions, and emergency response protocols.
	Appraising information	The capacity to contextualize medical recommendations relative to personal circumstances, evaluate source credibility, and critically weigh the risk-benefit profiles of treatment alternatives.
	Applying information	Practical adherence to medication protocols, informed treatment decision-making, and appropriate utilization of emergency services.
Disease prevention	Accessing information	The structured retrieval of information targeted at managing behavioral health risks (e.g., substance use, smoking), mitigating chronic stress, and navigating immunization or prophylactic screening schedules.
	Understanding information	The comprehension of the clinical rationale underlying prophylactic interventions, routine diagnostic screenings, and public health warnings regarding high-risk lifestyles.
	Appraising information	The capacity to autonomously determine the clinical necessity of preventive medical consultations and critically filter health risk reporting within mass media.

	Applying information	The capacity to make autonomous decisions regarding immunization protocols and internalize guidance from support networks or media vectors to mitigate personal health threats.
Health promotion	Accessing information	The capacity to identify structural opportunities for physical activity, optimal nutrition, and psychological well-being, alongside fostering health-conducive environments within one's occupational or community sphere.
	Understanding information	The comprehension of nutritional profiling on consumer goods, peer-led wellness guidance, and expert recommendations regarding mental health maintenance.
	Appraising information	The systemic evaluation of macro-level living conditions, longitudinal lifestyle choices, and daily habits on personal longevity and aggregate well-being.
	Applying information	Active engagement in physical regimens, direct modification of personal living environments, and participation in community-driven interventions aimed at optimizing long-term health.

Table S2

Modern Interpretation of Andragogical and Transformative Principles in adult education

Core Principle (Knowles, 1980)	Contemporary Conceptualization (2025)	Pedagogical Application in Health Literacy
The Need to Know (The "Why")	Explicit alignment between curriculum content and tangible, real-world outcomes (Seetharaman et al., 2025).	Transparent, problem-focused learning objectives integrated within modular interventions.

Self-Concept (Autonomy)	Learners as self-directed agents demanding executive control over learning pathways (Ampler et al., 2024).	Flexible, modular, and self-paced instructional formats.
Prior Experience (Experience as a Resource)	Mobilization and critical processing of personal biographical experiences (Seetharaman et al., 2025).	Scenario-based reflections and interactive discussions to dismantle health stereotypes.
Readiness to Learn	Cognitive motivation peaks when addressing immediate, highly relevant life challenges (Ampler et al., 2024).	Context-specific instruction offering direct problem-solving utility.
Orientation to Learning (Practical Focus)	Strong preference for practical, problem-based architectures over decontextualized theory (Ampler et al., 2024).	Simulation exercises, case-based learning, and actionable community projects.
Motivation to Learn (Intrinsic Motivation)	Intrinsic cognitive drivers (e.g., agency, well-being) outweigh extrinsic compliance incentives (Bano & Hou, 2025).	Visible progress tracking and reflective feedback mechanisms fostering autonomous agency.
